# Percutaneous Valvular Closure Followed by TAV-in-TAV Intervention during a Single Procedure in order to Treat a Severe Paravalvular Leak after Performing TAVI in a Bicuspid Aortic Stenosis

**DOI:** 10.1155/2019/4825607

**Published:** 2019-04-15

**Authors:** Georgios Tzimas, Eric Eeckhout, Panagiotis Antiochos, Christan Roguelov, Stephane Fournier, Brahim Harbaoui, Pierre Monney, Olivier Muller

**Affiliations:** ^1^Department of Cardiology, University Hospital of Lausanne (CHUV), Rue du Bugnon 46, 1005 Lausanne, Switzerland; ^2^Department of Cardiology, Hospices civils de Lyon, hôpital de la Croix-Rousse, 103 Grand Rue de la Crx Rousse, 69004 Lyon, France

## Abstract

In an era where transcatheter aortic valve implantation (TAVI) indications and utilization are expanding beyond high-risk patients, paravalvular leak remains the intervention's Achilles heel. Effective reduction of paravalvular leak is important in order to ensure an optimal clinical outcome. We present here the first case report in which percutaneous valvular closure using Amplatzer plugs followed by a TAV-in-TAV intervention during the same procedure managed to resolve a severe paravalvular leak with haemodynamic instability, after TAVI for a bicuspid aortic stenosis.

## 1. Introduction

As the indications for and utilization of TAVI are expanding beyond high-risk patients [1], paravalvular leak (PVL) remains the intervention's Achilles heel, as it has a negative impact on both short- and long-term prognoses [2–7]. Although several studies had demonstrated that TAVI constitutes a feasible safe intervention in patients with bicuspid aortic stenosis (AS), PVLs were described as one of the intervention's greatest weakness, as the total incidence of PVLs seems to be higher than in patients with tricuspid AS [8, 9]. Percutaneous closure as well as TAV-in-TAV intervention consists an effective procedure in order to improve PVL severity and symptoms. We present here the first case report of a severe PVL following TAVI, treated with percutaneous valvular closure using Amplatzer plugs and TAV-in-TAV intervention, both performed during a single procedure.

## 2. Case Report

A 79-year-old man with a medical history of hypertension, stage G3b chronic renal insufficiency, and interstitial lung disease presented with a four-month history of progressive dyspnoea (NYHA III).

**Figure 1:**
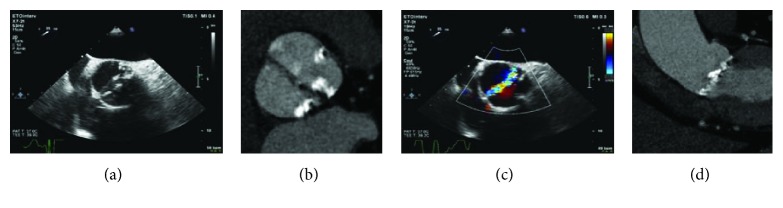
(a, b) Transthoracic echocardiography showing a bicuspid aortic valve. (c, d) Multidetector computed tomography scan revealing a large aortic valve annulus (annular area: 726.9 mm^2^, perimeter: 93.38 mm, long axis: 33.9 mm, and short axis: 26.2 mm) with severe aortic annular calcification.

Transthoracic echocardiography showed a bicuspid aortic valve (BAV) (Figures 1(a) and 1(b)) with severe, paradoxical, low-flow, low-gradient (mean gradient 26 mmHg) AS, a calculated aortic orifice area of 0.78 cm^2^, and a preserved left ventricular ejection fraction of 55%. A coronary angiogram revealed no significant epicardial coronary stenosis. Aortic root assessment was completed by multidetector computed tomography (MDCT), which revealed severe aortic annular calcification, with an aortic valve calcium score of 10133 AV and a large aortic valve annulus (Figures 1(c) and 1(d)). After using the three multiplanar reformation planes, measurements were derived from the area as well as the circumference of the virtual basal ring (mean diameter: 30.4 mm, annular area: 726.9 mm^2^, perimeter: 93.38 mm, long axis: 33.9 mm, and short axis: 26.2 mm). MDCT did not reveal any calcification extending into the left ventricular outflow tract. Additional supra-annular measurement of the valve opening area at the level of the maximal calcification did not show a significant mismatch compared with the initial measurements. Based on his severe comorbidities, characteristics of frailty, and refusal of surgery, the heart team decided to perform a TAVI via a femoral approach.

**Figure 2:**
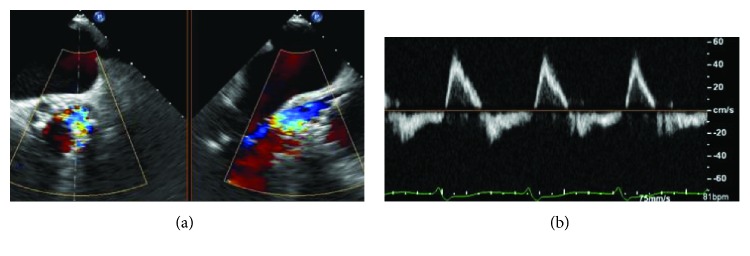
(a) Transoesophageal echocardiography showing a severe paravalvular leak after deployment of a CoreValve Evolut R 34 mm. (b) Pulsed wave Doppler in the descending aorta showing the presence of a consistent holodiastolic retrograde flow.

Balloon aortic valvuloplasty was performed using a 28 mm × 4 cm Nucleus balloon (NuMED, NY). Given the aspect of the valve marked by a large annulus with severe annular calcification and the eventual benefit of a valve resheathing and optimised repositioning, a CoreValve Evolut R 34 mm (Medtronic Inc., MN, USA) was implanted. After valve deployment, fluoroscopy and transoesophageal echocardiography (TEE) (Figures 2(a) and 2(b)) revealed a PVL due to (i) suboptimal valve expansion because of interference with the valvular ring by nodular calcifications and (ii) low positioning of the aortic valve prosthesis. The patient showed signs of haemodynamic instability, necessitating the initiation of inotropic support.

**Figure 3:**
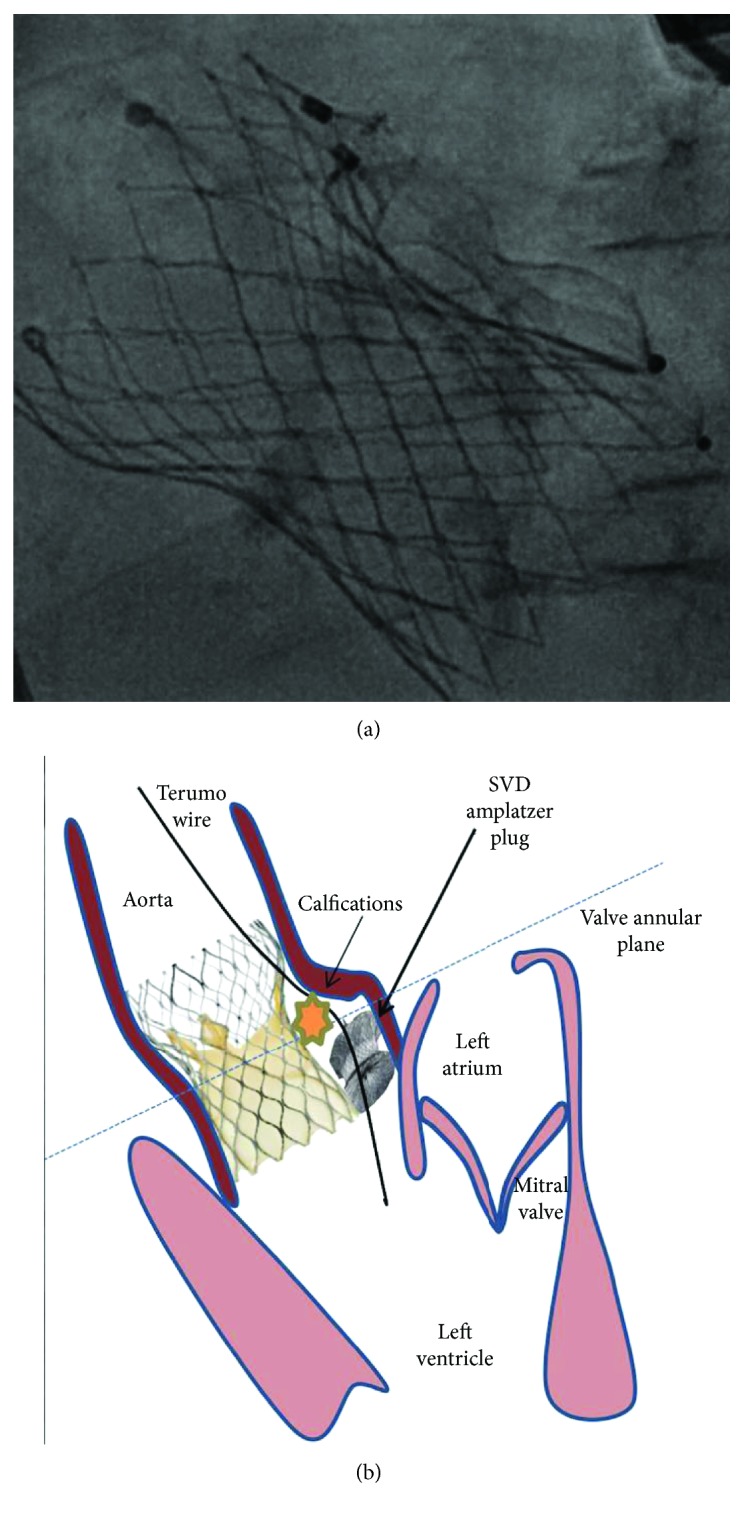
(a) Fluoroscopy with complete deployment of the two Amplatzer plugs. (b) A PVL was caused by the aortic prosthesis being set too low and its poor apposition against the heavily calcified commissure of the native aortic valve. A Terumo wire was pushed through the Evolut R valve's struts and then two SVD Amplatzer plugs were implanted. Due to persistent PVL after plug implantation, a further TAV-in-TAV intervention was performed. PVL: paravalvular leak; TAV: transcatheter aortic valve.

Our initial approach included postdilatation with the largest balloon available in our catheterization laboratory (28 mm × 4 cm Nucleus balloon (NuMED, NY)), attempting a better valve expansion and sealing the paravalvular space. However, fluoroscopy and TEE showed no improvement to the PVL (Supplementary 1). Among the possible strategies, percutaneous valvular closure using Amplatzer plugs constitutes a viable alternative in patients developing a PVL after TAVI [10, 11]. After an urgent discussion, the heart team decided to proceed with paravalvular implantation of a cardiac plug in an attempt to seal the PVL and improve the patient's haemodynamic status. After measuring the size of the leak by TEE, a Terumo wire (Terumo Interventional Systems, Somerset, New Jersey) was easily pushed through the aortic prosthesis struts, and the first 12 mm Amplatzer Vascular Plug II (AVPII) (AGA Medical Corp., Plymouth, Minnesota) was successfully implanted. Before detachment, coronary permeability was verified by coronary angiography. However, this failed to reduce the severity of the PVL. Following the same procedure as previously, we decided to implant a second 10 mm AVPII (Figures 3(a) and 3(b)) (AGA Medical Corp., Plymouth, Minnesota), which was positioned parallel to the first one. Again, fluoroscopy and TEE did not show any significant reduction in the PVL (Supplementary 2), and the patient remained haemodynamically unstable requiring continuous inotropic therapy.

**Figure 4:**
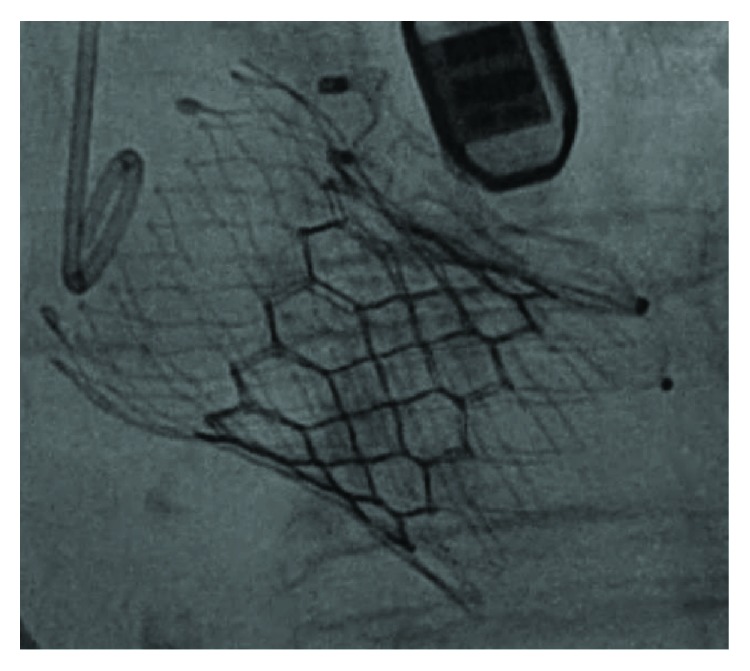
Fluoroscopy after complete deployment of the two Amplatzer plugs and the 29 mm EDWARDS Sapien 3.

**Figure 5:**
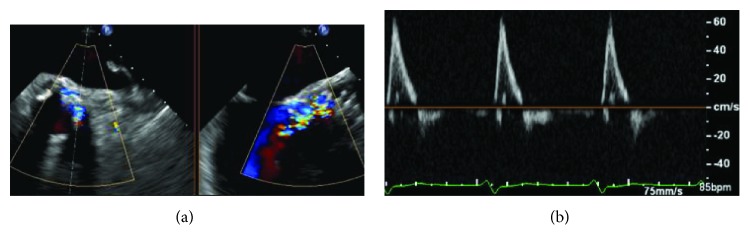
(a) Transoesophageal echocardiography revealing the sealing of the paravalvular leak and the presence of a mild residual aortic regurgitation after the deployment of a 29 mm EDWARDS Sapien 3. (b) Pulsed wave Doppler in the descending aorta showing an antegrade flow without diastolic flow reversal.

We decided to proceed with a TAV-in-TAV implantation as a rescue procedure, using a second aortic prosthesis (29 mm EDWARDS Sapien 3, Edwards Lifesciences, Irvine, California). This was deployed within the first valve (Figure 4), and the final outcome was the elimination of the AS, the sealing of the PVL, and the presence of a mild residual aortic regurgitation (Figures 5(a) and 5(b), Supplementary 3).

Nevertheless, a final aortic root angiography revealed a type A aortic dissection arising in the aortic sinuses and extending into the ascending aorta. The permeability of the coronary arteries was intact, and no pericardial infusion was observed. After a multidisciplinary discussion, a conventional surgical intervention was proposed to the family, but they refused this option. Continuation of all resuscitation therapy was abandoned, and the patient died three days later.

## 3. Discussion

Although new TAVI prostheses are designed to reduce the risk of residual aortic regurgitation, PVL after TAVI remains one of the most significant problems. Furthermore, challenging anatomies of bicuspid AS seem to be associated with a higher incidence of PVL following TAVI. Several studies have demonstrated that BAV is associated with more eccentric calcifications as well as with larger annulus. The asymmetry of the valve made of two unequal-sized leaflets can result in an extreme elliptical shape. This particular morphology together with the eccentric geometry of the aortic root increases the risk of uneven expansion of the valve. Despite the fact that percutaneous valvular closure using either Amplatzer plugs [10, 11] or TAV-in-TAV procedures [12, 13] is well described in the literature as reasonable strategies, to the best of our knowledge, this is the first case where both interventions were performed during a single procedure to minimise a severe PVL.

The present case illustrated the challenges of performing a TAVI in a bicuspid AS with a large valvular annulus. Large annulus and valve undersizing are well associated with PVL and prosthesis migration [14]. A recent study conducted by Attizzani et al. [15] showed a lower rate of procedural and device success, as well as longer procedural durations, among patients treated with a 31 mm CoreValve rather than other valve sizes (23, 26, and 29 mm). Higher rates of TAV-in-TAV bail-out procedures were also reported. The adoption of a sizing strategy based on a multimodal imaging assessment (CT, TEE) therefore seems crucial in order to avoid subsequent prosthesis migration due to the lack of anchoring support. In the present case, we believe that BAV morphology characterized by the fusion of two leaflets results in a potentially suboptimal valve sizing due to the difficulty to accurately identify the true annular plane. The valve undersizing as well as the bicuspid anatomy marked by an elliptic shape and affected by extreme and asymmetric calcification led to a suboptimal expansion of the first aortic prosthesis and its downward migration.

Unfortunately, the final stage of the procedure was further complicated by the presence of a type A aortic dissection which eventually led to patient death. Although the precise pathological mechanisms are not completely understood, the dilatation of the ascending aorta in BAV seems to be due to genetic as well as haemodynamic factors. The presence of a severe calcified aorta or a sharply angulated aortic arc as well as a dilated aorta predispose to a vascular complication. The probability increases when repeated endovascular gestures are performed during the intervention. Even though the latest studies have shown excellent results in treating bicuspid AS, especially with the use of new-generation devices, this category of patients was traditionally excluded from the large RCT due to the increased risk of PVL and vascular complications. Thus, these outcomes could further be improved by increased experience and the use of dedicated imaging modalities in the selection of patients.

In conclusion, this case demonstrated that a combined approach involving paravalvular Amplatzer plug implantation and a TAV-in-TAV intervention during the same procedure could be a potential option in order to reduce severe PVL associated with haemodynamic instability. However, we cannot exclude that a TAV-in-TAV implantation, as the sole intervention, might have been sufficient to seal the PVL.

## Supplementary Materials

Supplementary 1 Video 1: fluoroscopy showing a severe paravalvular leak after postdilatation of the CoreValve Evolut R 34 mm.

Supplementary 2 Video 2: fluoroscopy showing a persistent paravalvular leak despite the deployment of the two Amplatzer plugs.

Supplementary 3 Video 3: fluoroscopy revealing the sealing of the paravalvular leak and the presence of a mild residual aortic regurgitation after the deployment of a 29 mm EDWARDS Sapien 3.
